# Advanced operation of heated fluidic resonators via mechanical and thermal loss reduction in vacuum

**DOI:** 10.1038/s41378-023-00575-3

**Published:** 2023-10-10

**Authors:** Juhee Ko, Bong Jae Lee, Jungchul Lee

**Affiliations:** Department of Mechanical Engineering, Center for Extreme Thermal Physics and Manufacturing, Korea Advanced Institute of Science and Technology (KAIST), 34141 Daejeon, Korea

**Keywords:** Engineering, Sensors

## Abstract

For simultaneous and quantitative thermophysical measurements of ultrasmall liquid volumes, we have recently developed and reported heated fluidic resonators (HFRs). In this paper, we improve the precision of HFRs in a vacuum by significantly reducing the thermal loss around the sensing element. A vacuum chamber with optical, electrical, and microfluidic access is custom-built to decrease the convection loss by two orders of magnitude under 10^-4^ mbar conditions. As a result, the measurement sensitivities for thermal conductivity and specific heat capacity are increased by 4.1 and 1.6 times, respectively. When differentiating between deionized water (H_2_O) and heavy water (D_2_O) with similar thermophysical properties and ~10% different mass densities, the signal-to-noise ratio (property differences over standard error) for H_2_O and D_2_O is increased by 9 and 5 times for thermal conductivity and specific heat capacity, respectively.

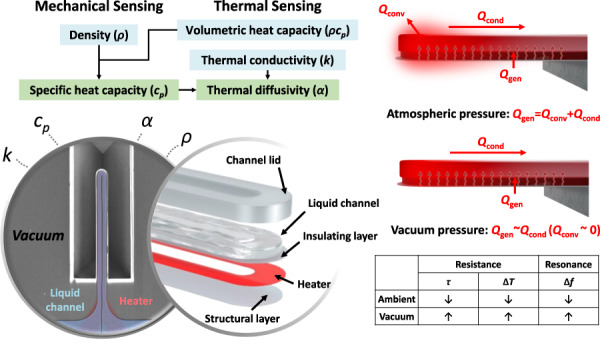

## Introduction

Thermophysical properties (e.g., thermal conductivity and heat capacity) of liquids are relevant to numerous applications, including thermal management of high-power electronic devices^1^, energy storage/harvesting systems^2^, and photothermal therapies^3^. There have been continuous efforts to measure thermophysical properties with microfluidics-integrated sensors^4–6^ that offer the advantages of fast response time and low sample consumption. Since most microfluidic platforms for thermophysical property measurements are not equipped with direct volume or mass measurements, their measurement results tend to be extensive and thus proportional to sample quantity. To integrate thermal sensing with gravimetric sensing metrology^7–10^, we recently introduced simultaneous, fast, and precise thermophysical property measurements using heated fluidic resonators (HFRs)^11^. With HFR, three intensive properties—thermal conductivity, specific heat capacity, and density—are simultaneously obtained by resistive heating/thermometry and resonant densitometry.

To increase sensitivity in thermal sensing including calorimetry^12,13^, thermophysical property measurement^14,15^, or single-cell analysis^16^, there have been various studies that reduce convection loss by creating a vacuum environment around the microfluidic channel. The effect of minimizing convection loss is more significant in micro/nanostructures due to the scaling effect of heat transfer^17^, which indicates that convection coefficients tend to increase with decreasing characteristic dimensions. Since our previous report^11^ is based on the atmospheric pressure operation of HFRs, the performance of these devices can be improved by decreasing convection loss in a vacuum.

In this paper, we propose such advanced operation of HFRs in a vacuum to improve their performance during thermophysical property measurements. Minimizing convection loss in a vacuum causes more heat generation within the integrated heater to be dissipated by conduction through the liquid sample inside the integrated channel. Therefore, the temperature responses of HFRs during pulse heating with fixed power levels show higher contrast for different liquids and enhance the sensitivity of thermophysical property measurements. With a custom-built vacuum chamber, we thoroughly characterize and compare measurement variables–resistance, and resonance frequency–in atmospheric and vacuum pressures. We confirm that the vacuum operation of HFRs improves the sensitivity as well as the signal-to-noise ratio for thermophysical property measurements.

## Methods

### Heated fluidic resonators

**Fig. 1:**
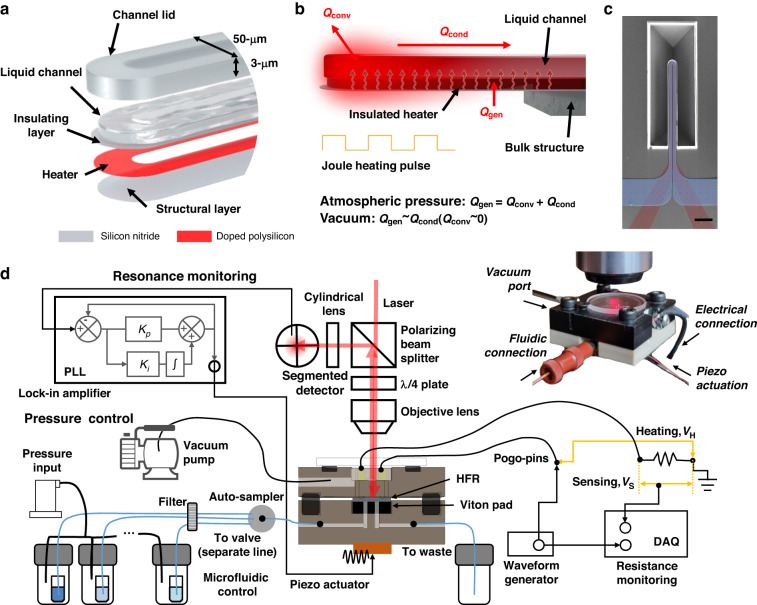
Sensor and system configuration. **a** 3D exploded view of a heated fluidic resonator (HFR). **b** Energy balance for HFR under joule heating in atmospheric and vacuum pressures. Convection loss becomes negligible in a vacuum. **c** Scanning electron micrograph of HFR with pseudocolored fluidic channel (blue) and heater (red). Scale bar is 100 µm. **d** A schematic illustration of the overall experimental setup and a photograph of the custom vacuum chamber for HFR with electrical and fluidic connections and vacuum port for advanced thermophysical property measurements with HFR in vacuum

HFRs are 500-µm long, 50-µm wide (16-µm of each channel), and 4-µm thick (3-µm channel height) suspended structures (MC516H, Life Analytical). The approach for mass sensing follows the procedure of previous liquid density measurements using a channel resonator^18–20^. As shown in Fig. 1a, HFRs are multilayered structures, where the thickness of each solid layer is optimized considering mass sensitivity and manufacturability. A doped and patterned polysilicon layer serves as a Joule heating electrode, and a silicon nitride layer is a fluidic channel structure and an electrical insulation layer between the channel and the heater. For thermal sensing, resistance is monitored in the time domain as resistive thermometry while a heating pulse is applied to the electrode, where an increase in resistance corresponds to an increase in temperature^21^. Steady-state resistance differences between the on and off states of the heating pulse and time constants of resistance at the rising edge are extracted to characterize the temperature response. Considering 30 ms of a pulse heating time as used previously^11^ and the increased time constant (~1 ms in atmospheric pressure) of HFR due to slowed thermal diffusion in a vacuum, pulse heating with 80-ms width and 160-ms period (50% duty) is used. A 3-mW amplitude and 3-mW DC offset are applied, which corresponds to 3 °C modulation at an average temperature of 27 °C in vacuum. Heat generation by Joule heating along the bottom heater surface is balanced with the sum of convection and conduction loss through the solid structure and liquid channel, as shown in the heat transfer diagram of the HFR (Fig. 1b). Different liquids inside the HFR change the conduction loss through the channel, resulting in a distinct temperature response at a fixed level of heating power. Figure 1c shows a scanning electron micrograph of the HFR with an integrated heater pseudocolored in red and a suspended fluidic channel pseudocolored in blue.

### Experimental setup with vacuum chamber

Thermophysical property measurement in a vacuum is performed with a custom vacuum chamber (Fig. 1d) that can control the environment to reduce the convective heat transfer coefficient around HFRs. The chamber is designed with fluid ports to transport liquid samples into the sensor, electrical connections for Joule heating and resistance measurements, and a transparent glass window that allows a laser to measure the resonance frequency (FC-2HVac, Life Analytical). To actuate the HFR, a piezoelectric chip (TA0505D024W, Thorlabs) is externally placed under the vacuum chamber. The chamber has an internal volume of ~0.2 mL and is connected by a vacuum pump (HiCube80, Pfeiffer Vacuum). Upon Joule heating pulses by a waveform generator (34401A, Keysight), the voltage and current are recorded simultaneously by a data acquisition module (USB-6361, National Instruments) using a voltage divider circuit to measure the resistance change over time. Inside the vacuum chamber, two pogo-pins, which are electrically insulated with chambers, are in direct contact with corresponding electrode pads, which are wired to the readout circuitry outside the chamber to ensure a stable electrical connection with negligible contact resistance (<0.1% of HFR resistance). The laser is focused on and reflected from the free end of the HFR and is monitored by a photodetector (S3096-02, Hamamatsu) to measure the resonance frequency. A phase-locked loop in a lock-in amplifier (HF2LI, Zurich Instruments) is used to record the resonance frequency in the time domain. A pressure regulator (ITV-0030, SMC) with an autosampler (MXX778, Rheodyne) is used to deliver liquid samples from small containers inside pressurized vials (50 kPa) into the HFR through 1/32-inch OD Teflon tubing. Microfluidic fittings (P-771, Upchurch) are used to connect the chamber with tubing. Details for the data acquisition have also been explained in the Supporting Information of our previous paper^11^.

### Important variables

Δ*R* is the electrical resistance (*R*) difference between the highest and lowest levels at the steady state during square pulse heating with a 50% duty cycle. *τ* is the characteristic time response extracted by fitting resistance changes with the exponential decay function ($R=A{{\rm {e}}}^{-\left(t-{t}_{0}\right)/\tau }+B$), where *A, B*, and ${t}_{0}$ are constants. Δ*f* is the difference in the time-averaged resonance frequency (*f*) under forced oscillation between the air-filled (*f*_0_) and specific liquid-filled HFR, simultaneously obtained with resistance monitoring.

## Results and discussion

### Characterization in vacuum

**Fig. 2:**
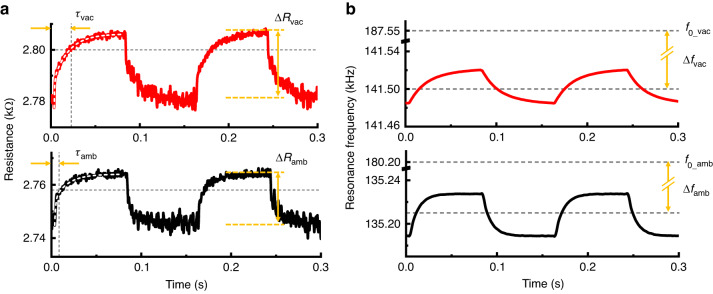
Simultaneous resistance and resonance measurements. **a** Resistance and **b** resonance frequency changes of HFR filled with the ethanol–water binary mixture (50:50 in volume) under 3-mW repeated heating pulses in atmospheric and vacuum pressures (~10^3^ and 10^−4^ mbar, respectively). Gray dashed lines in **a** show 63% of steady-state levels which match with time constants. Gray dashed lines in **b** show the time-averaged resonance frequency of air-filled HFR and sample-filled HFR, with their differences denoted by Δ*f*_vac_ in vacuum and Δ*f*_amb_ in atmospheric pressure

A comparison of the resistance (*R*) of HFR filled by an ethanol–water 50:50 binary mixture under atmospheric (~10^3^ mbar) and vacuum pressures (10^−4^ mbar) is shown in Fig. 2a. Similar to the higher baseline of *R* in a vacuum environment, the resistance difference (Δ*R*) with the same heating power is higher in a vacuum due to the reduced convective heat loss. Another feature of operation in the vacuum environment is a long time constant (*τ*) due to slowed thermal diffusion. The distinct thermal behavior differences between the two environments occur due to the significant difference in the convective heat transfer coefficient for the HFR, which is expected to be ~1000 W/m^2^ K based on a comparative analysis of spatial temperature mapping between Raman thermometry and finite element analysis (FEA, Fig. S1). Such high heat transfer coefficients for microscale devices have been reported in theoretical^17^ and experimental^22–24^ works. In our recent reports^25^, we have characterized the temperature response in a vacuum by using FEA, as illustrated in Figs. S2 and S3.

Figure 2b shows the comparison of resonance frequency (*f*) simultaneously measured with Fig. 2a. The slowed thermal diffusion shown in Fig. 2a also leads to a reduced time response of *f* in vacuum. Resonance spectra of HFR filled with a 50% ethanol-water binary mixture in atmospheric and vacuum pressure environments are illustrated in Fig. S4, where the *f* is upshifted (~6.4 kHz) and the quality factor (*Q*-factor)^26,27^ is increased (~5.9 times, Fig. S5) in a vacuum because of reduced mechanical damping from the air, which increased the oscillation amplitude by a factor of ~2. Due to increased *f* and oscillation amplitude^28^, heat transfer is enhanced in a vacuum. In fact, heat transfer is reduced overall because the decrease in density of the surrounding medium that suppresses heat transfer exceeds the increase in dynamic oscillation. The stability of *f* represented by the Allan deviation^29^ is compared between atmospheric and vacuum pressures, as shown in Fig. S6, where the minimum value of the Allan deviation in a vacuum is decreased to one-fifth of the level recorded in atmospheric pressure.

### Calibration of thermophysical properties

**Fig. 3:**
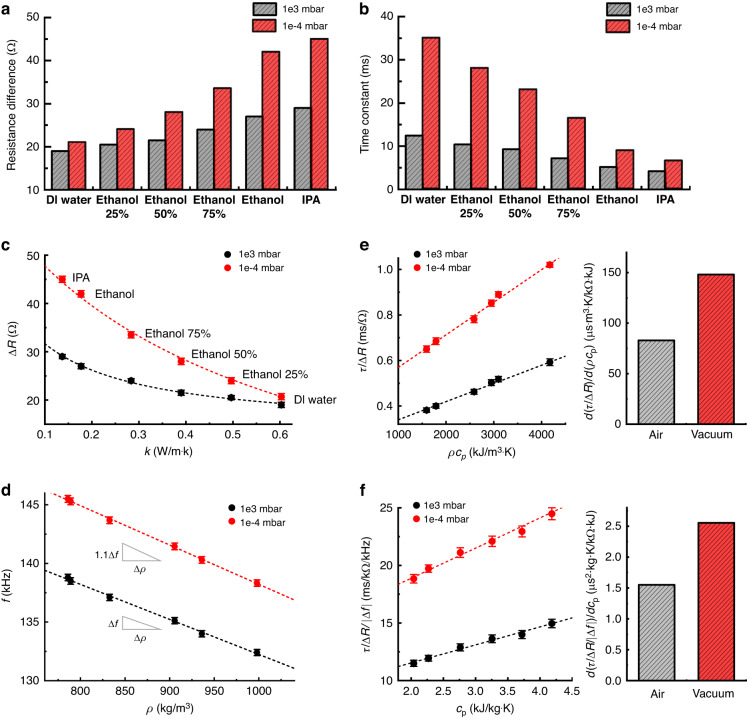
Thermal property measurements. **a** Resistance differences and **b** time constants extracted from the transient resistance response for six different liquid samples (deionized water, ethanol, ethanol–water mixtures, and isopropanol). **c** Resistance differences (∆*R*) of HFR filled with various liquid samples as a function of thermal conductivity (*k*). **d** Resonance frequencies of HFR as a function of liquid mass density. Density sensitivities are indicated by gray-colored triangles positioned near the data points. **e** Time constant (*τ*) divided by ∆*R* (*τ*/∆*R*) as a function of volumetric heat capacity (*ρc*_p_, left) and its slope (right), and **f** *τ/*∆*R* divided by resonance frequency change (|∆*f*|) as a function of specific heat capacity (*c*_p_, left) and its slope (right). All dashed lines are curve fits of measurement results by following theoretical relationship or dimensional analysis. All error bars represent standard errors from 800 measurements. All data are compared under atmospheric and vacuum pressures (~10^3^ and 10^−4^ mbar, respectively)

The changes in Δ*R* and *τ* of HFRs filled with six different liquids are observed in both vacuum and atmospheric pressure environments, as depicted in Fig. 3a and b, respectively. The increase in Δ*R* depends on the total thermal resistance of the HFR, which is related to the thermal conductivity (*k*) of the filled liquids. For measuring deionized water and ethanol binary mixtures, increasing the concentration of ethanol (which has a lower *k* than water) increases Δ*R*. In addition, the increase in *τ* depends on the total thermal capacitance of the HFR, which is related to the thermal mass or volumetric heat capacity (*ρc*_p_) of the filled liquids. Thus, increasing the concentration of ethanol reduces *τ* because ethanol has a lower *ρc*_p_ than water. Considering the equivalent electrical circuit of a heat transfer system within the HFR (Fig. 1b), the total thermal resistance is calculated as the harmonic mean of the thermal resistances of heat conduction through a liquid sample and a solid structure of the HFR, while the total thermal capacitance is the sum of the thermal capacitance of the liquid and the solid structure. Thus, the total thermal capacitance is more dependent on the thermal properties of liquids than the thermal resistance, which results in a larger difference in *τ* than Δ*R*.

To ensure measurement reliability, physical relationships between the measurement variables and thermophysical properties are obtained by dimensional analysis^11^^,^ as depicted in the Supporting Information. From this analysis, we found that (1) Δ*R* = *A*/(*k*‒*B*) + *C* for *k*, (2) *τ*/Δ*R* = *aρc*_p_ + *b* for volumetric heat capacity (*ρc*_p_), and (3) *τ*/Δ*R*/*|*Δ*f*| = *dc*_p_ + *e* for specific heat capacity (*c*_p_), where *A, B, C, a, b, d*, and *e* are calibration coefficients (see Fig. S7 for details). These coefficients are extracted by curve fitting (Levenberg‒Marquardt algorithm) of measurement variables, as shown in Fig. 3c–f, where the local standards of *k* and *ρc*_p_ by the hot-disk method^30,31^ (TPS 3500, Hot Disk Instruments) with density (*ρ*) reference from the literature^32^ are used. To enhance the precision of measurement, the average value and standard error from 800-pulse repetitions are obtained for both calibration and subsequent measurements.

The sensitivity of *k* is defined as the 1^st^ derivative of a fitting function for Δ*R* (Fig. 3c) and is represented as *d*Δ*R*/*dk* in Fig. S8. Compared to the decreasing sensitivity with a high *k* range in atmospheric pressure, a relatively higher sensitivity (4.1 times in the range of deionized water) is retained in a vacuum. Moreover, the sensitivity of *ρ*, *ρc*_p_, and *c*_p_ can be represented by their slope from linear function fits, where the sensitivity of *ρ* with *f* (Fig. 3d), *ρc*_p_ with *τ*/Δ*R* (Fig. 3e), and *c*_p_ with *τ*/Δ*R*/*|*Δ*f*| (Fig. 3f) are increased by 1.1, 1.8, and 1.6 times in vacuum, respectively. The sensitivity difference between *ρc*_p_ and *c*_p_ occurs due to the multiplied *ρ*. For a microfluidic resonator, the *ρ* sensitivity is proportional to the mechanical stiffness of the structure (*k*_m_) divided by the effective mass^33^ (*m*_eff_). In a vacuum, the pressure difference between the surroundings and inside the channel can induce bulging^34^ in the direction of channel thickness, leading to an increase in *k*_m_ and *m*_eff_. Among those factors, an increase in *k*_m_ is more dominant in the characteristics of the system and leads to an improvement in *ρ* sensitivity. On the other hand, frequency noise is related to *f*, *k*_m_, and the *Q*-factor. In a vacuum, a decrease in the density of the surrounding air reduces viscous damping, which increases the *Q*-factor and reduces inertial mass, which increases *f*. Since normalized frequency noise is reduced by the increase in *k*_m_, *Q*-factor, and *f*, theoretical *ρ* resolution is improved by considering both *ρ* sensitivity and normalized frequency noise. The *c*_p_ sensitivity is proportional to *τ*/Δ*R*/*|*Δ*f*|. All of the variables *τ*, Δ*R*, and |Δ*f*|, are increased in a vacuum, and the dominant increase in *τ* enhances *c*_p_ sensitivity. Since the temperature response of the HFR is affected by both thermophysical properties and the convective heat transfer coefficient, the convective heat transfer coefficient cannot be extracted solely by monitoring temperature changes over time. By comparison of *ρc*_p_ sensitivity, where the heat transfer coefficient is the sole factor to change, with FEM (Fig. S3a), the heat transfer coefficient at 10^−4^ mbar is calculated to be 25 W/m^2^ K (gray-dashed line in Fig. S3b).

### Thermophysical property measurements in vacuum

**Fig. 4:**
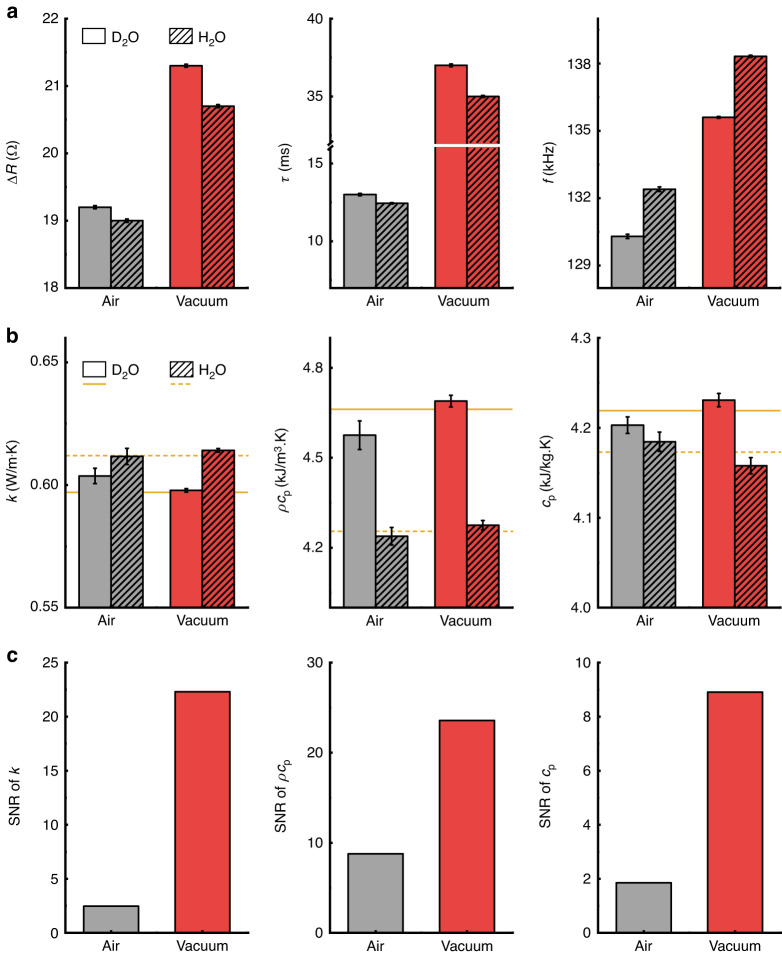
Performance metrics comparison in air and vacuum. **a** Resistance difference (∆*R*), time constant (*τ*), and resonance frequency (*f*) of HFR filled with heavy water (D_2_O) and deionized water (H_2_O). **b** Measurement results of thermal conductivity (*k*), volumetric heat capacity (*ρc*_p_), and specific heat capacity (*c*_p_) of D_2_O and H_2_O. **c** Signal-to-noise ratio (SNR) of thermophysical property measurements. All data are compared in atmospheric and vacuum pressures (~10^3^ and 10^−4^ mbar, respectively)

To compare the signal-to-noise ratio (SNR) or sensitivity of *k*, deuterium oxide (D_2_O) was chosen as a test material (Fig. 4a). D_2_O and deionized water (H_2_O) exhibit comparable *k*, falling within a range where the sensitivity to *k* is low in ambient measurements. The higher value of Δ*R* in D_2_O occurs due to its lower *k* than H_2_O, while the higher *τ* of D_2_O occurs due to its larger *ρc*_p_, and the lower *f* occurs due to its larger *ρ* than H_2_O. Thermophysical properties are extracted using the calibration curve in Fig. 3 and compared with local standards represented as dark-yellow solid (D_2_O) and dashed lines (H_2_O) in Fig. 4b. Since the noise of measurement variables shows less than ~10% difference between both environments, higher sensitivity in vacuum lowers the standard errors in thermophysical property measurements, as shown in Fig. 4b. Despite these improved measurement sensitivities in the vacuum, a comparison between the measurements and local standards does not always show a consistently better match (e.g., *k* and *c*_p_ of H_2_O). The difference between the measurement results and the local standards is mainly attributed to the fact that the properties of H_2_O are near the upper limit of the liquid properties used for calibration. If SNR is defined as the difference between H_2_O and D_2_O divided by the standard error, then the vacuum shows enhanced SNRs of those three variables (9 times for thermal conductivity, 3 times for volumetric heat capacity, and 5 times for specific heat capacity) compared to the air environment, as shown in Fig. 4c. Table 1 summarizes the sensitivity, resolution, and accuracy data for three thermophysical property measurements of D_2_O. Figure S9 illustrates that our measurement volume, as well as the relative error between the local standards and measured values, reached a minimum when compared to previous reports on thermal property measurements using small liquid sample volumes. The only exception to this trend occurred when using the 3-omega method with a 1 µL sample volume, which requires a frequency sweep and long measurement time.

**Table 1:** Performance metrics of thermophysical property measurements with HFR

		Sensitivity		Resolution		Accuracy (%)
*k*	Air	43.9 (EtOH)	[Ω m K/W]	0.003	[W/m K]	–
		8.8 (D_2_O)		0.009		98.87
	Vacuum	76.1 (EtOH)		0.001		–
		36.3 (D_2_O)		0.003		99.86
*ρc*_p_	Air	82.9	[µs m^3^ K/kΩ kJ]	0.143	[kJ/m^3^ K]	98.14
	Vacuum	149.1		0.106		99.42
*c*_p_	Air	1.6	[µs^2^ kg K/kΩ kJ]	0.203	[kJ/kg K]	99.62
	Vacuum	2.6		0.159		99.72

With differences in *c*_p_ and *ρc*_p_, *k* has a nonlinear relationship with Δ*R*; thus, the sensitivity and resolution of *k* are obtained for two different liquids (at the highest and lowest ranges within our samples). Resolution is calculated by the measurement of standard error. Accuracy is obtained by comparison between measurements of D_2_O with HFR and local standards with a hot-disk method. For *k*, the accuracy for other liquids, including ethanol (EtOH), is expected to be better than that for D_2_O because of the higher sensitivity but similar noise level

## Conclusion

In this paper, we improve the performance of thermophysical property measurements using our recently reported heated fluidic resonators (HFRs) by operating them in a vacuum. The vacuum chamber is constructed to decrease convection loss around the HFR, resulting in a two-orders-of-magnitude reduction in the convective heat transfer coefficient from ~1000 W/m^2^ K (at ~10^3^ mbar, atmospheric pressure) to 25 W/m^2^ K (at 10^−4^ mbar, partial vacuum pressure). The reduced thermal loss and slowed thermal diffusion in vacuum increase the differences in resistance and time constants recorded for the various liquid samples. As a result, the measurement sensitivities for all three intensive thermophysical properties reported here are increased by 4.1 times for thermal conductivity, 1.8 times for volumetric heat capacity, and 1.6 times for specific heat capacity. Based on their similar thermophysical properties, using H_2_O and D_2_O allowed us to show that the signal-to-noise ratios of thermal conductivity and specific heat capacity are increased by 9 and 5 times, respectively. Thus, we highlight the sensing performance of HFR with the lowest measurement error among the real-time thermophysical property measurement methods inside microfluidics and the importance of thermal loss management in micro/nanoscale sensors.

### Supplementary information

Supporting Information
