# Supplementary material for: Advanced operation of heated fluidic resonators via mechanical and thermal loss reduction in vacuum
Source: Microsyst Nanoeng. 2023 Oct 10;9:127. doi: 10.1038/s41378-023-00575-3 (PMC10564801; doi:10.1038/s41378-023-00575-3)
Supplement: Supplementary file 1 — Supporting Information [file 41378_2023_575_MOESM1_ESM.pdf]

Supporting Information

Advanced Operation of Heated Fluidic Resonators via Mechanical and Thermal Loss Reduction in Vacuo

Juhee Ko, Bong Jae Lee & Jungchul Lee¶

Center for Extreme Thermal Physics and Manufacturing, Department of Mechanical Engineering, Korea Advanced Institute of Science and Technology (KAIST), 34141, Daejeon-si, Korea

¶*Corresponding Author*

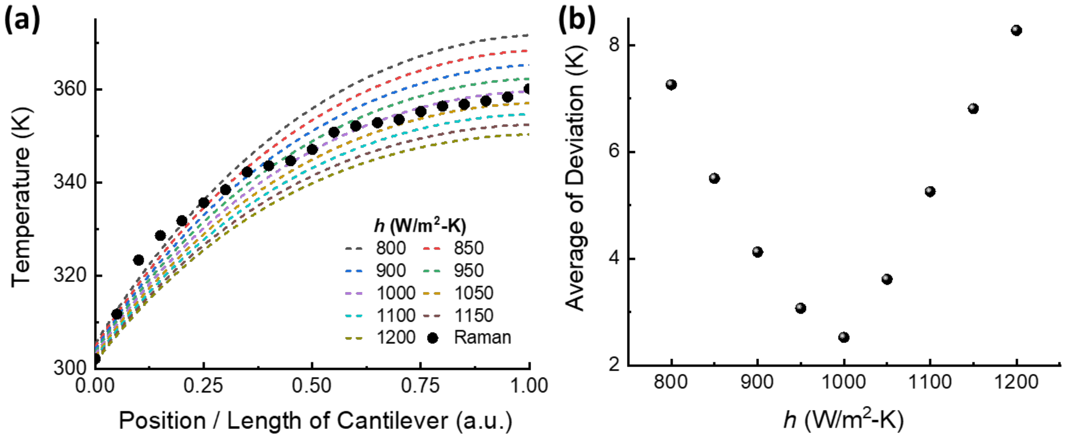

Figure S1. (a) Temperature distribution of HFR along channel length at different convective heat transfer coefficients from FEM analysis and measurement results using Raman thermometry[S1–S3]. The larger deviation in the clamped base is due to the undercut of the silicon nitride insulation layer. (b) The average temperature difference between FEM analysis and Raman thermometry as a function of convective heat transfer coefficients. It is best matched with the case of $h = 1000$ W/m^2·K.

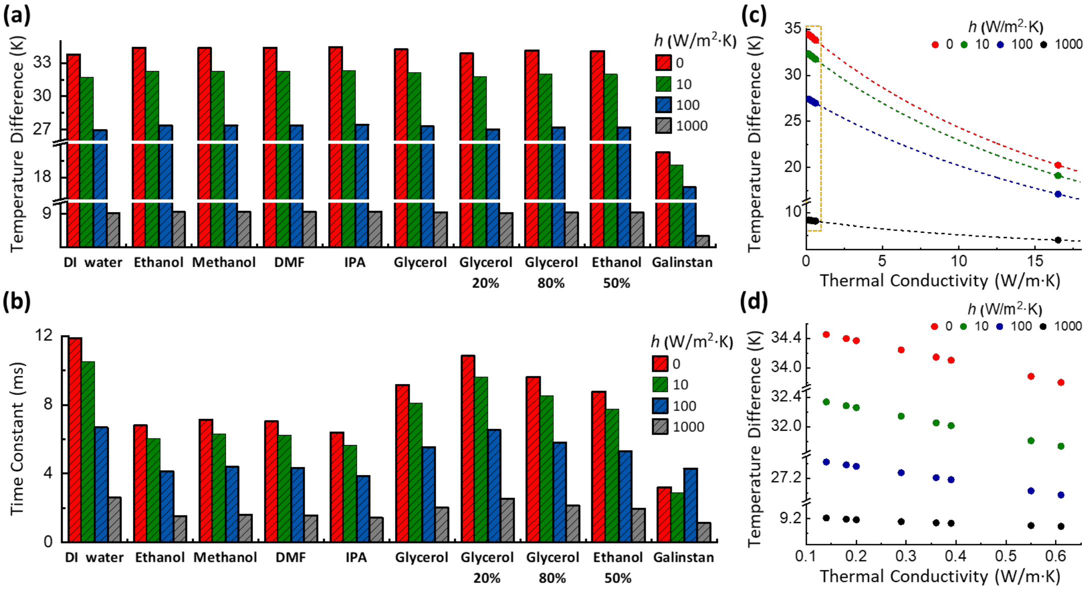

Figure S2. (a) Temperature differences and (b) time constants of HFR filled with various liquid samples under a 10-mW heating pulse with different convective heat transfer coefficients of 0, 10, 100, and 1000 W/m^2·K. (c) Temperature differences of HFR filled with various samples as a function of thermal conductivity and (d) their zoomed-in graph. All data are obtained from transient FEM heat transfer simulation.

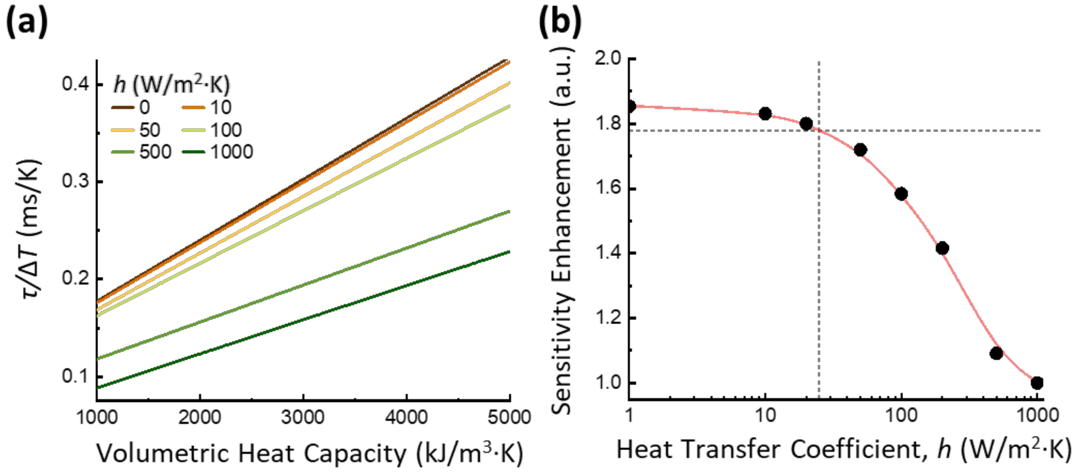

Figure S3. (a) Time constant divided by temperature difference as a function of volumetric heat capacity for various convective heat transfer coefficients. (b) Sensitivity enhancements calculated by a slope in (a), compared to the heat transfer coefficient at atmospheric pressure (1000 W/m^2·K). Although comparative analysis of temperature distribution between experiments and FEA can also provide the heat transfer coefficient as in Figure S1, spatial mapping of Raman thermometry in every partial vacuum level is a practical challenge (time-consuming).

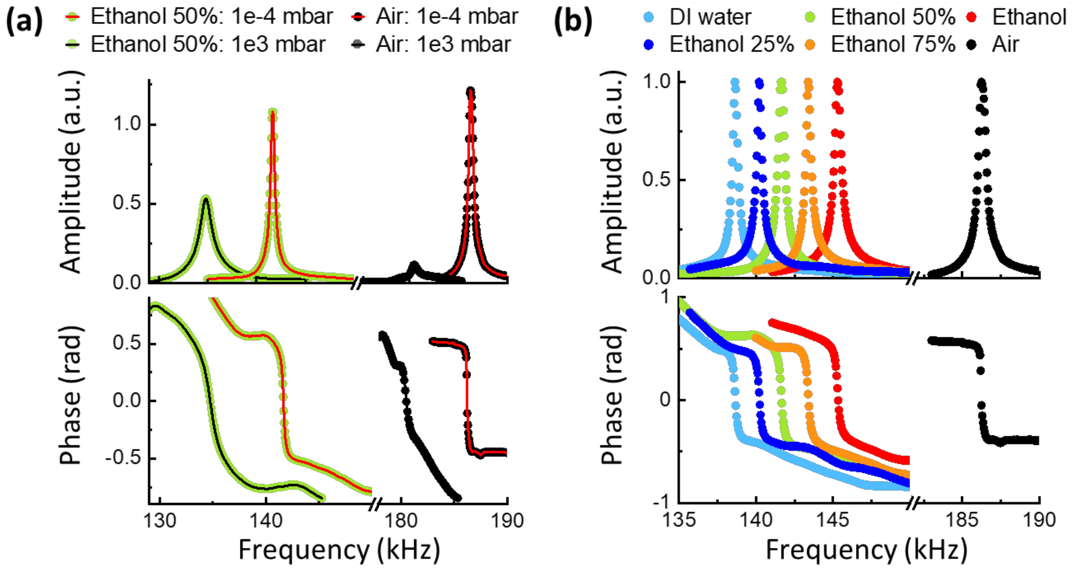

Figure S4. (a) Amplitude spectra of HFR filled with air and ethanol-water binary mixture (50:50 in volume) in atmospheric and vacuum pressures (~10^3 and 10^{-4} mbar, respectively). (b) Amplitude and phase spectra of HFR filled with various ethanol-water binary mixtures in vacuum pressure.

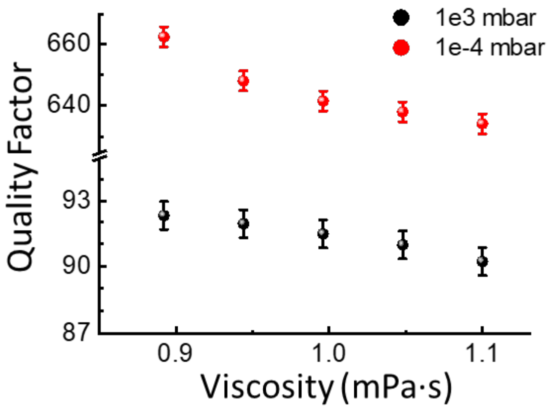

Figure S5. Quality factor changes of HFR as a function of liquid viscosity in atmospheric and vacuum pressures (~10^3 and 10^{-4} mbar, respectively).

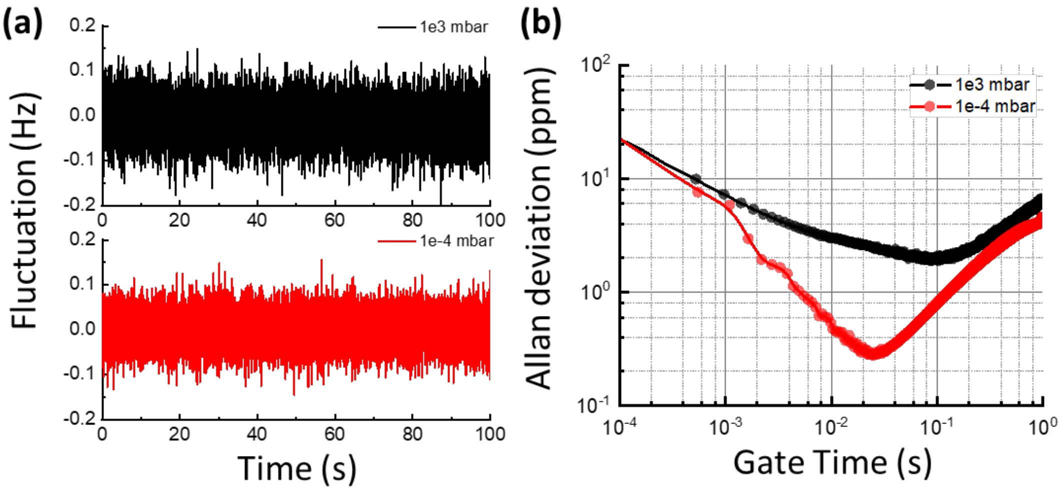

Figure S6. (a) Resonance frequency fluctuation of HFR obtained with a sampling rate of 1.8 MSa/s during 100 s. Considering the time duration of one heating pulse is 80 ms, 100 s is sufficient time to get measurement variables of more than 1000 pairs. (b) Allan deviation of resonance frequency fluctuation showing the minimum value of 0.28 ppm at the gate time of 0.025 s for 10^{-4} mbar and 1.83 ppm at the gate time of 0.105 for ~10^3 mbar.

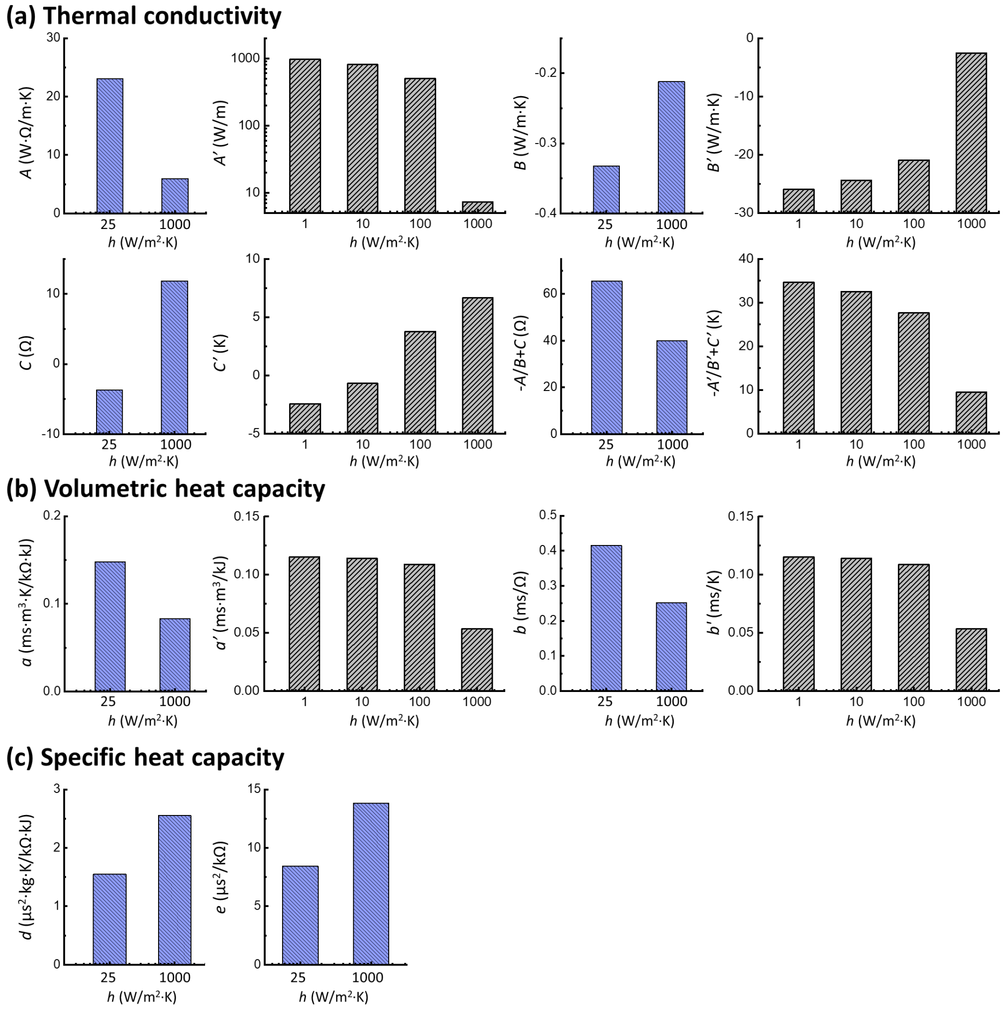

Figure S7. (a) Curve fitting coefficients for thermal conductivity measurement from experiments (blue-colored) and FEA (gray-colored, $\Delta T = A'/(k-B')+C'$). (b) Curve fitting coefficients for volumetric heat capacity measurement from experiments and FEA ($\tau/\Delta R = a'\cdot\rho c_p + b'$) where τ is time constant and ρc_p is volumetric heat capacity. (c) Curve fitting coefficients for specific heat capacity measurement from experiments.

During curve-fittings to find coefficients, upper or under limits are considered by their physical meaning. For example, B must have a negative value so that the ΔR does not diverge to infinity at the positive k. For thermal conductivity, trends of y-intercept (when $k = 0$), $-A/B+C$, show an increase with vacuum, owing to decreasing thermal loss through ambiance during the specific power modulation. Although trends of horizontal asymptote, C (when $k\rightarrow\infty$) are considered to have the same in vacuum and air, curve fits based on the limited thermal conductivity range of general liquids show differences between them. On the other hand, vertical asymptotes closer to the y-axis in air affect the higher sensitivity in the low k range (gaseous phase in general). These physically incomprehensible two features indicate that the extension of the calibration curve over the measurement range will not match with the samples other than the liquid phase.

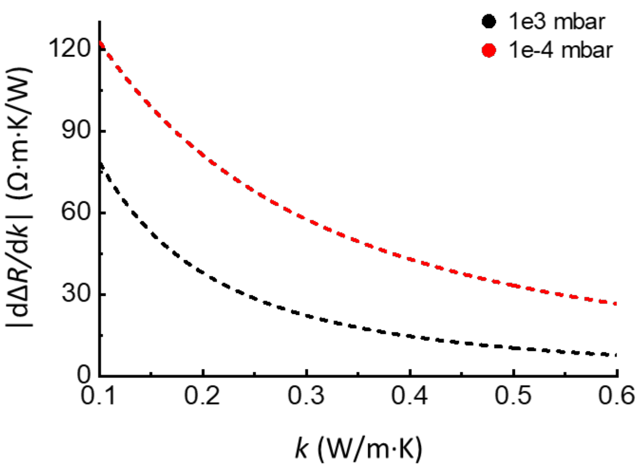

Figure S8. Slopes (1st order derivatives of fitting function) of resistance difference (ΔR) as a function of thermal conductivity (k) in atmospheric and vacuum pressure (~10^3 and 10^{-4} mbar, respectively).

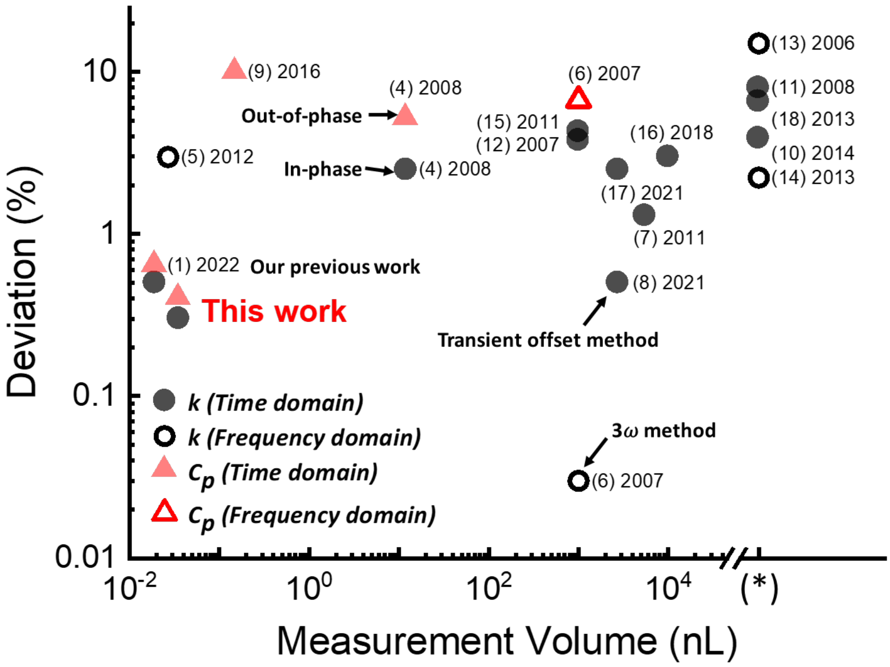

Figure S9. The average deviation of thermal conductivity and heat capacity by using HFR in vacuum environment, compared to the state-of-the-art of thermophysical properties measurements with micro thermal sensors from our previous result[S1] and the other references[S4–S18]. References in (*) use methods that are hard to define the measurement volume. HFR with a different length from the previous work is used in this work. Studies so far have been developed by random introduction of new metrologies rather than improving performance.

Dimensional analysis of thermophysical properties measurement

Considering a governing equation for the temperature (T) along the length (L) of HFR with a constant power (Q) as also described in the previous paper[S1],

$$kA\frac{\partial^2 T}{\partial x^2} - hP(T - T_\infty) - \rho c_p A\frac{\partial T}{\partial t} + Q/L = 0 \tag{1}$$

The dimensional functional equation can be written as

$$T - T_\infty = fn\left(T|_{x=0} - T_\infty, x, L, kA, hP, Q, t, \rho c_p\right) \tag{2}$$

where k is a thermal conductivity, A is a cross-sectional area, h is a convective heat transfer coefficient around the HFR, P is a perimeter, T_∞ is an ambient temperature, ρ is a density, c_p is a specific heat capacity, and t is a time. We have concluded with the following relationship for steady-state measurements,

$$k \propto \frac{1}{\bar{T} - T_\infty} \tag{3}$$

where h and A are considered constant and $\bar{T} = \int_{x=0}^{x=L} T(x)dx/L$. In a perfect vacuum state for $h = 0$, there is no need to consider h in a variable in dimensionless group. Although h in our system doesn't reach to zero, just reduced by two orders of magnitude, h can be considered as a constant for invariant vacuum level at each environment (both atmospheric and vacuum pressure environment). In addition, A is increased due to channel bulging by inside and outside pressure difference in vacuum, but it can also be considered as a constant for its invariance at both environments. Therefore, the resulting relationships from the dimensional analysis in the previous paper can be used same here.

Here, we can express k with measurement variable, resistance (R) and resistance difference (ΔR), since R is proportional to average temperature ($\bar{T}$) for relatively low temperatures (<600 K in our experiment).

$$k \propto \frac{1}{R - R_0}\left(= \frac{1}{\Delta R}\right) \tag{4}$$

For transient conditions, which leads to time constant $\tau = \rho c_p L^4/kA$,

$$\rho c_p \propto \tau k \text{ and } c_p \propto \tau k/\rho \tag{5}$$

Similarly, measurement variables such as resistance difference (ΔR) and electrothermal time constant (τ_R) from transient resistance response during heating, and resonance frequency shift (Δf) can be used to express ρc_p and c_p.

$$\rho c_p \propto \tau k \propto \frac{\tau_R}{\Delta R} \; and \; c_p \propto \tau k/\rho \propto \frac{\tau_R}{|\Delta f|\Delta R} \tag{6}$$

References for Supporting Information

1. Ko, J., Khan, F., Nam, Y., Lee, B. J. & Lee, J. Nanomechanical sensing using heater-integrated fluidic resonators. *Nano Lett.* **22**, 7768–7775 (2022).
2. Beechem, T., Graham, S., Kearney, S. P., Phinney, L. M. & Serrano, J. R. Invited article: Simultaneous mapping of temperature and stress in microdevices using micro-Raman spectroscopy. *Rev. Sci. Instrum.* **78**, 061301 (2007).
3. Lee, J. *et al.* Electrical, thermal, and mechanical characterization of silicon microcantilever heaters. *J. Microelectromech. Syst.* **15**, 1644–1655 (2006).
4. Choi, S. R. & Kim, D. Real-time thermal characterization of 12 nl fluid samples in a microchannel. *Rev. Sci. Instrum.* **79**, 064901 (2008).
5. Park, B. K., Yi, N., Park, J. & Kim, D. Note: Development of a microfabricated sensor to measure thermal conductivity of picoliter scale liquid samples. *Rev. Sci. Instrum.* **83**, 106102 (2012).
6. Choi, S. R., Kim, J. & Kim, D. 3ω method to measure thermal properties of electrically conducting small-volume liquid. *Rev. Sci. Instrum.* **78**, 084902 (2007).
7. Kuvshinov, D. *et al.* Thermal conductivity measurement of liquids in a microfluidic device. *Microfluid. Nanofluidics* **10**, 123–132 (2011).
8. Oudebrouckx, G., Vandenryt, T., Bormans, S., Wagner, P. H. & Thoelen, R. Measuring thermal conductivity in a microfluidic device with the transient thermal offset (TTO) method. *IEEE Sens. J.* **21**, 7298–7307 (2021).
9. Khan, M. F. *et al.* Heat capacity measurements of sub-nanoliter volumes of liquids using bimaterial microchannel cantilevers. *Appl. Phys. Lett.* **108**, 211906 (2016).
10. Kujawska, T., Secomski, W., Kruglenko, E., Krawczyk, K. & Nowicki, A. Determination of tissue thermal conductivity by measuring and modeling temperature rise induced in tissue by pulsed focused ultrasound. *PLoS One* **9**, e94929 (2014).
11. Zhu, S., Marcotte, M., Ramaswamy, H., Shao, Y. & Le-Bail, A. Evaluation and comparison of thermal conductivity of food materials at high pressure. *Food Bioprod. Process.* **86**, 147–153 (2008).
12. Choi, S. R. & Kim, D. Measurement of thermal properties of microfluidic samples using laser point heating thermometry. *Thermochim. Acta* **455**, 11–15 (2007).
13. Kuntner, J., Kohl, F. & Jakoby, B. Simultaneous thermal conductivity and diffusivity sensing in liquids using a micromachined device. *Sens. Actuators A, Phys.* **130–131**, 62–67 (2006).
14. Gauthier, S., Giani, A. & Combette, P. Gas thermal conductivity measurement using the three-omega method. *Sens. Actuators A, Phys.* **195**, 50–55 (2013).
15. Yi, N., Park, B. K., Kim, D. & Park, J. Micro-droplet detection and characterization using thermal responses. *Lab. Chip* **11**, 2378–2384 (2011).
16. Shrestha, R., Atluri, R., Simmons, D. P., Kim, D. S. & Choi, T. Y. A micro-pipette thermal sensing technique for measuring the thermal conductivity of non-volatile fluids. *Rev. Sci. Instrum.* **89**, 114902 (2018).

17. Oudebrouckx, G. *et al.* Single element thermal sensor for measuring thermal conductivity and flow rate inside a microchannel. *Sens. Actuators A, Phys.* **331**, 112906 (2021).
18. Shrestha, R. *et al.* Steady heat conduction-based thermal conductivity measurement of single walled carbon nanotubes thin film using a micropipette thermal sensor. *Rev. Sci. Instrum.* **84**, 034901 (2013).
19. Yarin, L. P. *The Pi-Theorem*. (Springer Berlin Heidelberg, 2012). doi:10.1007/978-3-642-19565-5.
